# Insights into the current state of knowledge, practice, and attitudes of physicians regarding gastrointestinal motility disorders in Egypt

**DOI:** 10.1186/s12876-024-03296-7

**Published:** 2024-07-04

**Authors:** Enaam Ali Al Mowafy, Marwa M. AboKresha, Sally Waheed Elkhadry, Mohamed Bassam Hashem, Ahmed Elganzory, Sayed Ahmed Sayed, Mohammad Almohamady Khaskia

**Affiliations:** 1 Tropical Medicine Department, Ain Shams University, Cairo, Egypt; 2 Internal Medicine Department, Assuit University, Assuit, Egypt; 3 Epidemiology and Preventive Medicine Department, National Liver Institute, Menoufia University, Menoufia, Egypt; 4 Endemic Medicine Department, Cairo University, Cairo, Egypt

**Keywords:** Gastrointestinal motility disorders, Education and training, Physicians’ understanding, Current understanding, Clinical practice, Clinicians’ attitudes

## Abstract

**Background:**

Gastrointestinal (GI) motility disorders are common in clinical settings, but physicians still lack sufficient understanding and effective management of these conditions.

**Methods:**

This research assessed Egyptian physicians’ knowledge, practices, and attitudes towards GI motility disorders. A cross-sectional survey employing a self-administered questionnaire was carried out among physicians in Egypt. The questionnaire addressed various aspects of physicians’ understanding, practices, and attitudes regarding GI motility disorders. Data analysis was conducted using descriptive statistics and presented as frequencies and percentages.

**Results:**

A total of 462 physicians took part in the study. Although nearly two-thirds of them knew about GI motility studies, a notable proportion lacked adequate knowledge about GI motility disorders. Notably, 84.2% correctly identified dysphagia as a critical symptom suggestive of an upper GI motility disorder. However, 13.4% incorrectly linked hematemesis with an upper GI motility disorder, and 16.7% expressed uncertainty. In terms of practice, around half of the participants encountered a small number of patients with GI motility disorders (less than 5 per week or even fewer). Only 29.7% felt confident in managing patients with motility disorders. Most participating physicians expressed a willingness to participate in training programs focused on motility disorders.

**Conclusions:**

This study underscores a knowledge gap among Egyptian physicians concerning GI motility disorders. It suggests the necessity of tailored education and training programs to improve their competency and practice in this domain.

**Supplementary Information:**

The online version contains supplementary material available at 10.1186/s12876-024-03296-7.

## Background

Gastrointestinal (GI) motility disorders such as gastroparesis, functional dyspepsia, enteric dysmotility, irritable bowel syndrome, and constipation have a substantial global impact, reducing quality of life and imposing significant burdens on health insurance systems [1, 2].

GI motility disorders manifest as unexplained symptoms affecting various parts of the GI tract. These include the upper oesophagal sphincter, oesophagal peristalsis, lower oesophagal sphincter, gastric emptying, small intestinal motility, colonic transit, colonic dysbiosis, and anorectal issues such as dyssynergia and anal sphincter defects. Distinguishing GI motility disorders from organic conditions (such as inflammatory or malignant diseases) heavily relies on patient history, which poses diagnostic challenges [3, 4].

GI motility disorders pose a diagnostic challenge. They manifest as unexplained symptoms affecting various parts of the GI tract, including the upper oesophagal sphincter, oesophagal peristalsis, lower oesophagal sphincter, gastric emptying, small intestinal motility, colonic transit, colonic dysbiosis, and anorectal issues such as dyssynergia and anal sphincter defects [3]. In clinical practice, the first step to assess GI symptoms is to exclude organic diseases, mainly if there are any concerning signs such as weight loss, bloody stool, abdominal masses, lymphadenopathy, or anemia [5]. Motility tests are usually performed for patients with persistent complaints associated with GI motility disorders, significantly affecting their quality of life, nutrition, social functioning, and work productivity, or occasionally elevating mortality risk [6, 7]. Furthermore, distinguishing GI motility disorders from organic conditions (such as inflammatory or malignant diseases) relies heavily on patient history [4].

Recent progress has brought forth sophisticated diagnostic instruments to evaluate GI motility and function [8, 9]. The International Working Group on Disorders of GI and Function strongly encourages the consistent use of these diagnostic tests to detect clinically relevant problems and guide treatment decisions [10, 11]. Despite advancements in this particular area, there remains a lack of overall agreement on the proper protocols for diagnosing and treating extremely severe gastrointestinal motility disorders. This absence of agreement has triggered debates among experts globally, leading to considerable variability in clinical practice [12]. Unfortunately, this circumstance may have increased the number of intestinal failures as a result of severe GI motility disorders [13].

In Egypt, similarly, despite numerous global publications, there is a limited emphasis on GI motility research. We hypothesized that insufficient awareness, ambiguity, and inconsistencies in defining and investigating GI motility disorders might contribute to physicians’ hesitancy in ordering GI motility studies. Consequently, Egyptian patients with such disorders are likely to be underdiagnosed. To comprehensively address this issue, we interviewed Egyptian physicians to explore their attitudes toward adult patients with GI motility disorders, evaluate their clinical practices, and pinpoint specific knowledge gaps in this domain.

## Methods

An observational cross-sectional study was conducted in Egypt to examine physicians’ knowledge, practice, and attitudes towards adult patients with GI motility disorders.

### Data collection

Physicians were invited to answer a questionnaire. The questionnaire was uploaded online (Microsoft Forms or Google Forms) to be self-employed for physicians to answer. They were invited through several social media platforms (Facebook, Twitter, and Whatsapp).

### Questionnaire structure

The questionnaire administered in this study comprised two primary sections. Part I gathered data related to socio-demographic characters of the study participant, including age, gender, residence, educational qualifications, specialty, and workplace details. Part II inquired into assessing participants’ knowledge, practices, and attitudes about GI motility disorders. It included questions about understanding and preferences regarding motility disorders, exploration of clinical practices, and evaluation of attitudes towards these conditions.

### Questionnaire validation

We ensured that our questionnaire achieved content validity and was well-prepared for further assessment in real-world settings. The following steps were taken: (1) Content Validation Form Preparation: The content validation form was developed to ensure the review panel understood their task clearly. This form served as a guide for evaluating the questionnaire. (2) Expert Review Group Selection: A review group of experts with specific knowledge of the subject matter was meticulously formed. The committee comprised seven professors: two gastroenterologists (MA and MAG), one public health expert (SWE), one specialist in endemic medicine (ME), and three tropical medicine professors (HMA, MAS, and IFM). (3) Pilot Testing and Cognitive Interviewing: Trained team members (EAS) administered the translated questionnaire to 20 participants. Participants’ understanding, readability, language usage, wording, cultural appropriateness of items, and clarity of response instructions were assessed during these interviews. Some interviewees encountered difficulties comprehending certain modified items. Additionally, participants provided valuable feedback on specific questions. (4) Final Version Approval: The questionnaire was refined based on insights from pilot testing and cognitive interviews. Subsequently, the researchers approved the final version, preparing it for field testing.

### Statistical analysis

Data were presented as mean ± SD for quantitative variables and percentages and frequencies for qualitative variables. Statistical tools from the Epi website were utilized to determine the sample size. The sample size calculation considered a 5% margin of error, a 95% confidence interval, and an estimated design effect (DEFF) of one (*n* = [DEFF*Np(1-p)]/[(d2/Z21-α/2*(N-1) + p*(1-p)]). Initially, the estimated minimum sample size was 384 participants. However, the actual collected sample size was 462 participants.

### Ethical consideration

The study obtained approval from the Ethics Committee of the National Liver Institute, Menoufia University, Egypt (IRB No: 00416/2022). It adhered to the International Ethical Guidelines for Epidemiological and Descriptive Studies.

## Results

### Study’s demographics and participant characteristics

Our study had a diverse pool of physicians. All Egyptians, the majority from Greater Cairo (35%). Males participated more than female physicians (56.9% to 43.1%). Physicians aged 30 to 34 were more than any other age group (37.2%). Most study participants held master’s degrees (30.3%) or MDs (33.3%), indicating high education and expertise. Participants averaged 8.45 years of specialty field experience, with 40.3% having fewer than five years. Most participants had diagnostic (52.4%) and therapeutic (29.7%) upper GI endoscopic experience (Table 1).

**Table 1:** Demographic data of the participants

		Number	Percent
Gender	Male	263	56.9
	Female	199	43.1
Age	25–29	99	21.5
	30–34	171	37.2
	35–39	102	22.2
	40–44	52	11.3
	45–49	17	3.7
	50–54	7	1.5
	55–59	7	1.5
	60 and above	5	1.1
Specialty	Gastroenterology	295	63.9
	Internal Medicine	89	19.3
	Others	78	16.9
Place of work	University Hospital	331	71.6
	Public Hospital	77	16.7
	Educational hospital	15	3.2
	Others	39	8.4
Last academic degree	MBBch	111	24
	Master	140	30.3
	MD	154	33.3
	Diploma & Fellowship	57	12.4
Type of health center working in	Primary	35	7.6
	Secondary	47	10.2
	Tertiary hospital	380	82.3
Years of experience in specialty	Mean ± SD Median (Min–max)	8.45 ± 6.6 7(0–50)	
Years of experience in your specialty	less than 5 years	186	40.3
	5–10	154	33.3
	10–15	70	15.2
	15–20	29	6.3
	20–25	12	2.6
	more than 25 years	11	2.3
Experience in endoscopy	Diagnostic upper GI endoscopy	242	52.4
	Diagnostic Colonoscopy	124	26.8
	Therapeutic endoscopy	137	29.7
	retrograde cholangiopancreatography [ERCP]	48	10.4
	Endoscopic ultrasound [EUS]	21	4.5
	Not applicable	178	38.5
Years of experience in endoscopy *N* = 289	Mean ± SD Median (min–max)	5.43 ± 5.25 4(0–35)	
	Egyptians	462	100
	Greater Cairo	162	35.1
	Lower Egypt (Delta region and North Egypt)	54	11.7
	Upper Egypt	90	19.5

### Participants’ knowledge of GI motility studies and symptoms

**Fig. 1:**
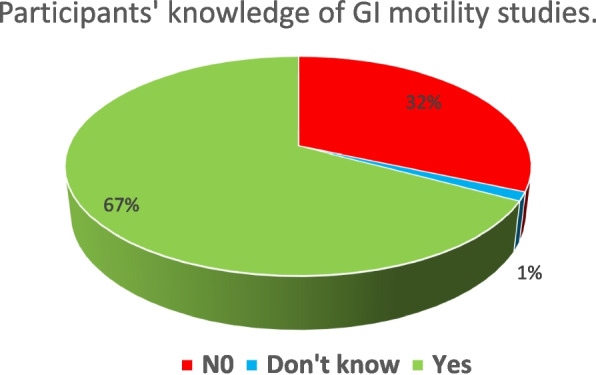
Participants’ knowledge of GI motility studies

Approximately 67.1% of participants indicated familiarity with GI motility studies, while 31.6% responded that they did not know, and only a small percentage (1.3%) were unsure. Dysphagia, odynophagia, reflux symptoms, non-cardiac chest pain, vomiting, eructation, and epigastric pain were recognized as symptoms of upper GI motility disorders by the majority of participants, with percentages ranging from 48.7% to 84.2%. Although not typically associated with upper GI motility disorders, hematemesis was identified as such by a small percentage (13.4%) of participants (Table 2, Fig. 1).

**Table 2:** Participants’ knowledge of GI motility studies

	N(%)	N(%)	N(%)
**Do you know what GI motility studies are?**	N0	I don’t know	Yes
	146(31.6%)	6(1.3%)	310(67.1%)
**Which symptoms make you suspect an upper GI motility disorder?**	**Not a symptom of upper motility disorder**	**Don’t Know**	**Symptoms of upper motility disorder**
Dysphagia	21(4.5%)	52(11.3%)	389(84.2%)
odynophagia	145(31.4%)	92(19.9%)	225(48.7%)
Reflux symptoms	76(16.5%)	51(11.0%)	335(72.5%)
Noncardiac chest pain	44(9.5%)	62(13.4%)	356(77.1%)
Vomiting	89(19.3%)	54(11.7%)	319(69.0%)
Eructation	86(18.6%)	68(14.7%)	308(66.7%)
Hematemesis^a^	323(69.9%)	77(16.7%)	62(13.4%)
Epigastric pain	157(34.0%)	58(12.6%)	247(53.5%)
**Which symptoms make you suspect a lower GI motility disorder?**	**Not a symptom of lower GI motility disorder**	**Don’t Know**	**Symptoms of lower GI motility disorder**
Constipation	5(1.1%)	64(13.9%)	393(85.1%)
Bloating	73(15.8%)	71(15.4%)	318(68.8%)
Diarrhea	64(13.9%)	72(15.6%)	326(70.6%)
Bloody diarrhea*	288(62.3%)	74(16.0%)	100(21.6%)
Incontinence	105(22.7%)	74(16.0%)	283(61.3%)
Need to strain	65(14.1%)	53(11.5%)	344(74.5%)
Sense of obstruction	59(12.8%)	83(18.0%)	320(69.3%)
Long periods between motions	55(11.9%)	67(14.5%)	340(73.6%)

^a^Not a GI motility disorder

**Fig. 2:**
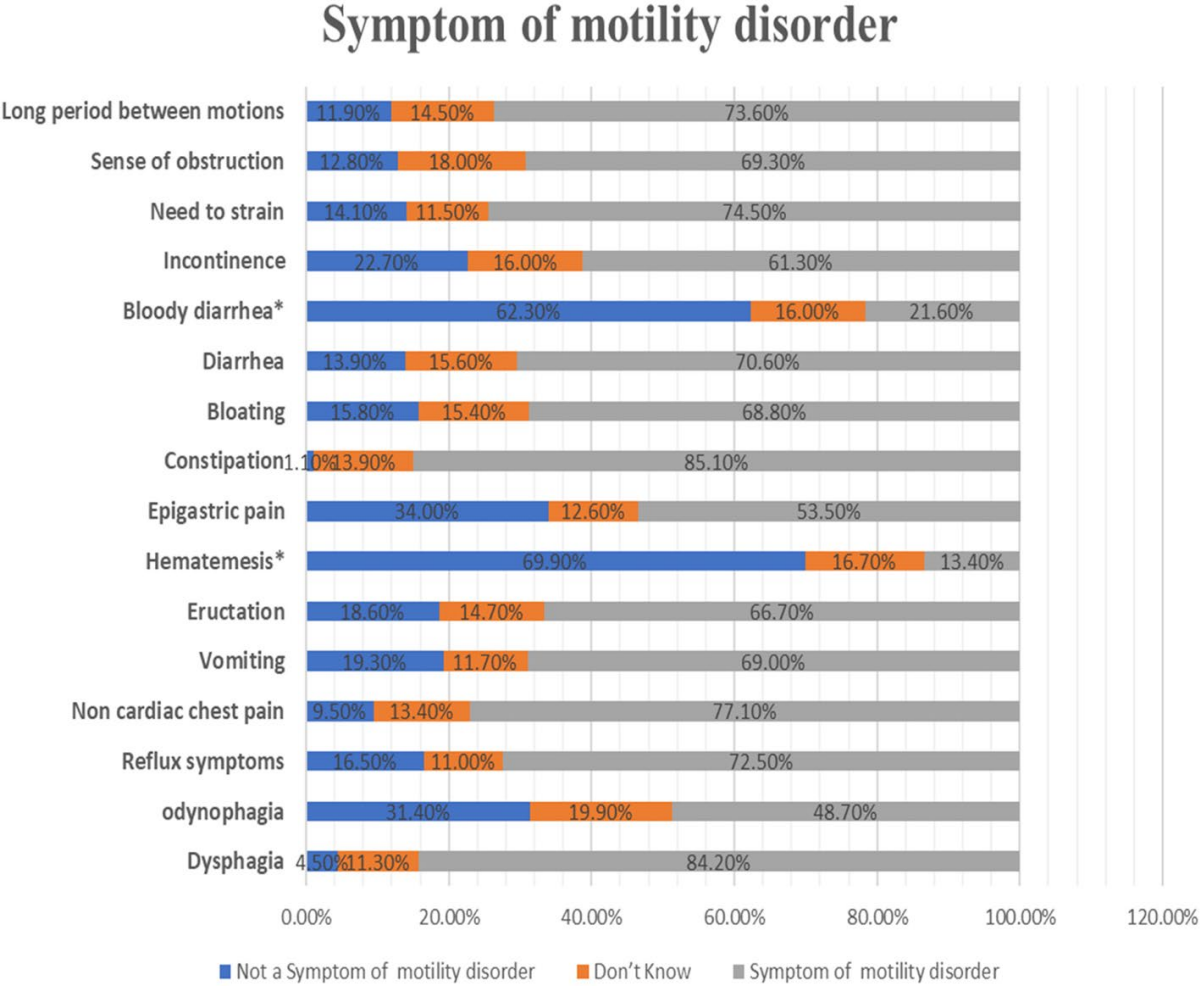
Participants’ knowledge of GI motility symptoms *Not a GI motility disorder.*Long period between motions = constipation

Symptoms such as constipation, bloating, diarrhea, bloody diarrhea, incontinence, the need to strain, a sense of obstruction, and long periods between motions were identified as symptoms of lower GI motility disorders. Notably, while constipation was recognized as a symptom by the majority (85.1%) of participants, bloody diarrhea, which is not typically associated with lower GI motility disorders, was acknowledged by 21.6% of participants (Table 2, Fig. 2).

### Participants’ knowledge of GI motility diagnostic methods

A significant portion of participants (63.8%) emphasized the importance of endoscopy as a primary investigative tool for diagnosing various GI motility disorders. The study also revealed overwhelming agreement among participants (84.9%) regarding the indispensable nature of manometry in diagnosing GI motility disorders. Furthermore, participants collectively acknowledged other investigative modalities relevant to diagnosing GI motility disorders. Most (59.1%) recognized pH studies as crucial for assessing acid reflux and esophageal motility. Similarly, a notable percentage (67.1%) affirmed the importance of the barium study, indicating its value in highlighting structural abnormalities and functional issues within the GI tract. Scintigraphy garnered significant recognition, with 74.7% of participants acknowledging its relevance in diagnosing motility disorders and emphasizing its utility in evaluating gastric emptying and transit times. However, opinions regarding the utility of computed tomography (CT) were more diverse, with 65% agreeing or strongly agreeing on its importance, while 34.8% expressed neutral or disagreeing perspectives. This divergence suggests varying participant interpretations regarding CT’s role in diagnosing GI motility disorders. Additionally, endoscopic ultrasound emerged as a priority investigation modality, with 63.2% of participants recognizing its significance (Table 3).

**Table 3:** Knowledge about investigations, modalities, and diagnosis of different GI motility disorders

	Strongly disagree	Disagree	Neutral	Agree	Strongly agree
**Grade the following investigation modalities regarding their priority in diagnosing different GI motility disorders.**					
Endoscopy (Upper/Lower)	5(1.1%)	21(4.5%)	141(30.5%)	140(30.3%)	155(33.5%)
Manometry	-	2(0.4%)	68(14.7%)	91(19.7%)	301(65.2%)
pH study	3(.6%)	55(11.9%)	131(28.4%)	133(28.8%)	140(30.3%)
Barium study	5(1.1%)	18(3.9%)	129(27.9%)	180(39.0%)	130(28.1%)
Scintigraphy	6(1.3%)	45(9.7%)	207(44.8%)	138(29.9%)	66(14.3%)
Computed tomography of chest, abdomen & pelvis	21(4.5%)	94(20.3%)	175(37.9%)	125(27.1%)	47(10.2%)
Endoscopic ultrasound	20(4.3%)	106(22.9%)	176(38.1%)	116(25.1%)	44(9.5%)

### Participants practice for GI motility disorders

Many participants saw GI motility problems, with 24.7% seeing 1–5 and 24.7% seeing 6–10. This highlights the substantial caseload and the importance of effective diagnostic and management strategies. Also, most doctors (34.0%) who think a patient has a GI motility disorder first do endoscopy and imaging, then manometry. This demonstrates a standard, step-by-step method for diagnosing GI motility disorder in clinical practice.

**Fig. 3:**
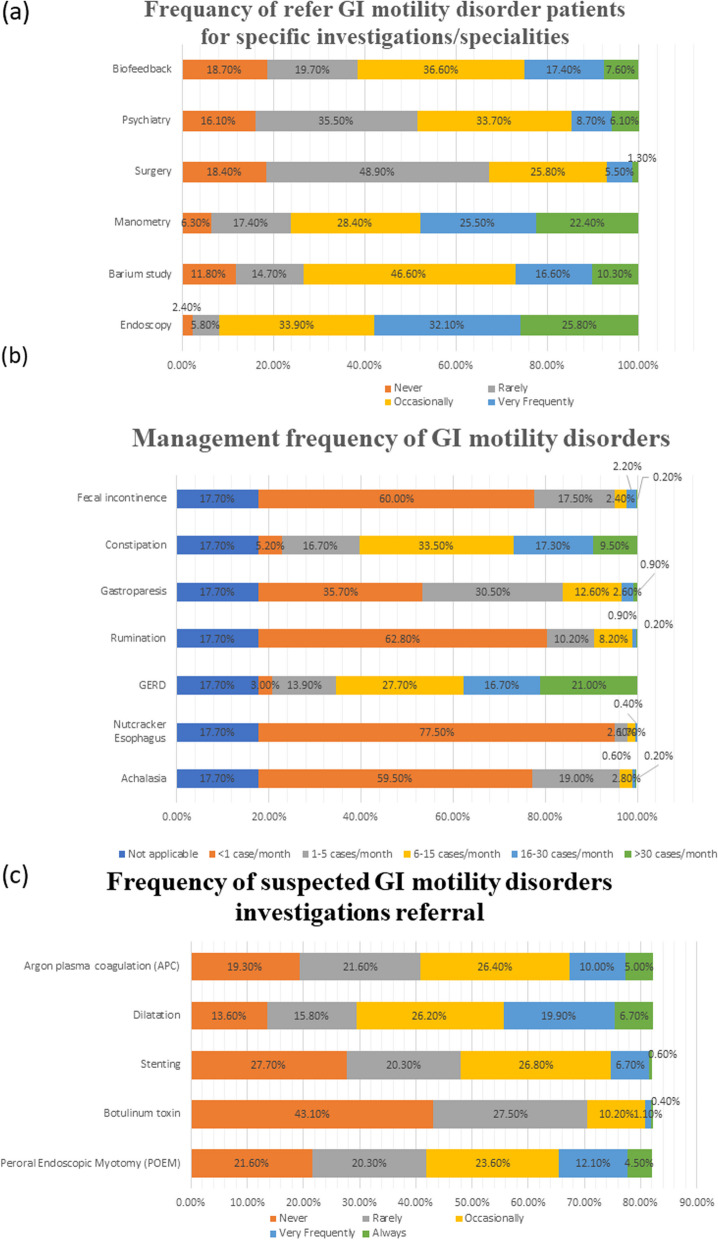
**a** GI motility disorder investigation referral frequency; **b** management frequency of GI motility disorders; and **c** frequency of suspected GI motility disorders investigations referral

The data demonstrates varying physician practices in referrals and interventions. Most participants refer patients to endoscopy (58.0%), whereas 6.8% and 7.4% suggest surgery and psychiatry. Similarly, 78.5% and 67.2% of physicians commonly manage achalasia and gastroparesis medically, while GERD and constipation receive more diverse management frequencies (Table 4, Fig. 3).

**Table 4:** Participants’ practice for GI motility disorders

	Not applicable	< 1 per week	1–5 per week	6–10 per week	11–15 per week	> 15 per week
**How frequently do you see patients with any GI motility disorders?**	22(4.7%)	114(24.7%)	114(24.7%)	46(10.0%)	11(2.4%)	16(3.5%)
		**Refer to a gastroenterologist**	**Perform endoscopy, imaging at first& then manometry**	**Treat and re-evaluate**	**Ask for manometry first**	**Other**
**From your practice of view, what do you do when you suspect a GI motility disorder? (overlapping answers)**		82(17.7%)	157(34.0%)	112(24.2%)	23(5.0%)	0(0%)
**How frequently do you refer patients for these interventions if you suspect a GI motility disorder (achalasia and gastroparesis)?**						
		**Never**	**Rarely**	**Occasionally**	**Very Frequently**	**Always**
Endoscopy		9(2.4%)	22(5.8%)	129(33.9%)	122(32.1%)	98(25.8%)
Barium study		45(11.8%)	56(14.7%)	177(46.6%)	63(16.6%)	39(10.3%)
Manometry		24(6.3%)	66(17.4%)	108(28.4%)	97(25.5%)	85(22.4%)
Surgery		70(18.4%)	186(48.9%)	98(25.8%)	21(5.5%)	5(1.3%)
Psychiatry		61(16.1%)	135(35.5%)	128(33.7%)	33(8.7%)	23(6.1%)
Biofeedback (In cases of defecatory disorders/constipation)		71(18.7%)	75(19.7%)	139(36.6%)	66(17.4%)	29(7.6%)
How frequently have you managed medically for any of the following conditions? * Not applicable *N* = 82(17.7%)						
	Not applicable	< 1 case/month	1–5 cases/month	6–15 cases/month	16–30 cases/month	> 30 cases/month
Achalasia	82(17.7%)	275(59.5%)	88(19.0%)	13(2.8%)	3(0.6%)	1(0.2%)
Nutcracker esophagus	82(17.7%)	358(77.5%)	12(2.6%)	8(1.7%)	2(0.4%)	–
GERD	82(17.7%)	14(3.0%)	64(13.9%)	128(27.7%)	77(16.7%)	97(21.0%)
Rumination	82(17.7%)	290(62.8%)	47(10.2%)	38(8.2%)	4(.9%)	1(.2%)
Gastroparesis	82(17.7%)	165(35.7%)	141(30.5%)	58(12.6%)	12(2.6%)	4(.9%)
Constipation	82(17.7%)	24(5.2%)	77(16.7%)	155(33.5%)	80(17.3%)	44(9.5%)
Fecal incontinence	82(17.7%)	277(60.0%)	81(17.5%)	11(2.4%)	10(2.2%)	1(.2%)
How frequently do you refer patients for these Interventions if you suspect motility disorder (achalasia, gastroparesis)? * Not applicable *N* = 82(17.7%)						
	Not applicable	Never	Rarely	Occasionally	Very Frequently	Always
Peroral endoscopic myotomy (POEM)	82(17.7%)	100(21.6%)	94(20.3%)	109(23.6%)	56(12.1%)	21(4.5%)
Botulinum toxin Injection	82(17.7%)	199(43.1%)	127(27.5%)	47(10.2%)	5(1.1%)	2(.4%)
Stenting	82(17.7%)	128(27.7%)	94(20.3%)	124(26.8%)	31(6.7%)	3(.6%)
Dilatation	82(17.7%)	63(13.6%)	73(15.8%)	121(26.2%)	92(19.9%)	31(6.7%)
Argon plasma coagulation (APC)	82(17.7%)	89(19.3%)	100(21.6%)	122(26.4%)	46(10.0%)	23(5.0%)

### Attitude of the participants about motility disorder

A significant proportion of hospitals lack dedicated motility units or machines, with 56.9% of participants reporting their absence. The reasons cited for this absence vary, including financial constraints, lack of expertise, and perceived lack of necessity due to nearby units. Despite these challenges, participants are strongly inclined to participate in research studies on motility disorders, with 71.2% expressing interest. Additionally, the overwhelming majority (77.9%) of participants affirm the importance of diagnosing GI motility disorders, highlighting the recognition of these conditions as significant health concerns. Furthermore, the belief in the benefits of early diagnosis is prevalent, with 79.7% of participants acknowledging the potential benefits to patients. However, perceived barriers to motility practice, including the complexity of the branch, lack of experts, and expensive investigations, suggest ongoing challenges in effectively addressing GI motility disorders (Table 5).

**Table 5:** Attitude of the participants about motility disorder

		Frequency	Percent
**Do you have a motility unit/machine in your hospital?**	No	263	56.9
	I don’t know	44	9.5
	Yes	155	33.5
**Causes of not having a motility unit/machine in your hospital**	Financial	108	23.4
	Not needed as there is a nearby unit	16	3.5
	No available experts	62	13.4
	I don’t know	9	1.9
	Of no use	6	1.3
**Are you interested in participating in research studies in motility studies?**	No	47	12.6
	Not Sure	60	16.1
	Yes	265	71.2
**Do you think it is essential to diagnose GI motility disorders**?	Yes	360	77.9
**Do you think that the patient will benefit from early diagnosis of a GI motility disorders?**	Not Sure	4	.9
	Yes	368	79.7
**What do you think are the barriers to GI motility practice?**			
**Difficult branch or not well understood**	Disagree	42	11.5
	Neutral	79	21.7
	Agree	152	41.8
	Strongly Agree	91	25.0
**Lack of experts**	Disagree	18	4.9
	Neutral	54	14.6
	Agree	172	46.6
	Strongly Agree	125	33.9
**No available investigation tool**	Disagree	25	6.7
	Neutral	67	18.1
	Agree	124	33.4
	Strongly Agree	155	41.8
**Expensive investigations**	Disagree	15	4.0
	Neutral	73	19.7
	Agree	145	39.1
	Strongly Agree	138	37.2
**To the physicians it is not financially rewarding like endoscopic procedures**	Disagree	45	12.3
	Neutral	102	27.8
	Agree	154	42.0
	Strongly Agree	66	18.0
**How are you comfortable when dealing/reading manometry report?**	Not Applicable (I don’t receive any reports)/ Not comfortable	149	43.3
	Neutral	119	34.6
	Comfortable	76	22.1
**How are you comfortable when dealing with a patient with motility disorder?**	Not Applicable	239	70.3
	Comfortable	101	29.7
**If there will be training about motility disorders, what would you like it to be about? And how do you suspect and diagnose motility disorders?**			
**How to manage motility disorders medically**	Disagree	15	4.0
	Agree	357	96.0
**Endoscopic management of motility disorders**	Disagree	43	11.6
	Agree	329	88.4
**How to read a manometry report/ topography**	Disagree	20	5.4
	Agree	352	94.6
**Investigations rather than manometry (e.g., radiological)**	Disagree	39	10.5
	Agree	333	89.5

## Discussion

The field of GI tract motility is dynamic and promising, with significant progress made over the past century, particularly in the last five decades. This progress is attributed to integrating various aspects, including electrophysiology, smooth muscle physiology, flow dynamics, neurohormonal physiology, and pharmaceutical research [14]. Interest in GI motility science surged in Egypt after adopting third-space endoscopic techniques in 2019, following the introduction of manometry devices in the 1990s [15].

This study represents a pioneering effort in Egypt and the broader Middle East to assess physicians’ knowledge, attitudes, and practices regarding GI motility studies. A total of 462 Egyptian physicians participated actively by answering the study questionnaire. The participant characteristics revealed a diverse demographic profile. According to the Association of American Medical Colleges, 56.9% of the participants were male, consistent with global trends, as males constitute over 80% of gastroenterologists [16]. Our study also underscores the diverse expertise and backgrounds of the participants, enriching the study’s insights and perspectives. One-third of the participants belonged to the 35–39 age group, with a significant portion falling within the 25–29- and 35–39-year brackets. Gastroenterologists dominated the study, constituting nearly two-thirds of the participants, while most were affiliated with university hospitals. Qualification-wise, around a third held either an MD, with a similar proportion possessing diplomas or fellowships. Specialty experience ranged widely from 0 to 50 years, with a mean of 8.45 ± 6.6, with 40% having less than 5 years of experience. Conversely, endoscopy experience ranged from 0 to 35 years, with a mean of 5.43 ± 5.25 (Table 1).

Our study indicates varying levels of knowledge and recognition among participants regarding GI motility studies and symptoms associated with upper and lower GI motility disorders. Dysphagia, a distressing oesophagal symptom, poses diagnostic challenges, particularly when functional causes are implicated after excluding organic factors [17]. Notably, vomiting is a hallmark symptom of gastroparesis [18], underlining the intricate relationship between GI motility and symptomatology. Moreover, gastroesophageal reflux disease (GERD) manifests through a spectrum of symptoms, including chest pain, dysphagia, odynophagia, epigastric pain, and nausea [19], mirroring a broader spectrum of motility disorders [20]. In our study, nearly 84.2% identified dysphagia as an essential symptom indicating an upper GI motility disorder, followed by 77.1% for non-cardiac chest pain, 72.5% for odynophagia, and 69% for vomiting as an upper GI motility disorder symptom.

Primary constipation is a significant subset of chronic constipation, often resulting from neuromuscular incoordination and frequently overlapped by dyssynergic defecation [21]. Regarding diarrhea, after excluding the presence of alarm symptoms such as bleeding, malnutrition, nocturnal diarrhea, and positive family history of colorectal cancer, dysmotility and functional causes should be investigated to diagnose irritable bowel disease with diarrhea and functional diarrhea [22]. In our study, 85.1% identified chronic constipation as an essential symptom indicating a lower GI motility disorder, followed by 74.5% for the need to strain, 70.6% for diarrhea, and 86.8% for bloating as lower GI motility disorder symptoms (Fig. 2).

GIT bleeding is a red flag of organic causes like inflammation, ulcers, varices, arteriovenous malformations, or malignancy [3]. Surprisingly, 13.4% said hematemesis is a sign of upper GI motility problems, while 16.7% didn’t know. While 16% didn’t know, 21.6% mistakenly replied that reduced GI motility problems cause bloody diarrhea. Nearly one-third of interviewees were unfamiliar with GI motility disorders (Table 2).

High-resolution manometry is well-known as the gold standard for diagnosing oesophagal motor dysfunction [23], especially when combined with impedance sensors [24]. pH studies are the most suitable, advanced method to diagnose GERD and are considered the gold standard [25]. Barium studies remain an essential diagnostic test in patients with motility disorders since they can identify structural lesions such as strictures and help identify significant motility disorders [26]. Gastric scintigraphy is a standard diagnostic tool for gastric dysmotility [27]. Our study findings suggest that participants generally recognize the importance of various investigation modalities in diagnosing GI motility disorders, with endoscopy, manometry, pH study, and scintigraphy particularly emphasized. Nearly two-thirds strongly agreed, and an additional 29.7% agreed that manometry is very important in the diagnosis of different GI motility disorders. However, a lesser percentage showed agreement for other investigations. One third strongly agreed, and an additional 28.8% agreed that pH studies are important in diagnosis, with nearly the same percent for barium studies. Unfortunately, due to wrong beliefs or lack of scientific basis, one-third strongly agreed that CT and abdominal ultrasound play a role in diagnosing GI motility disorders (Table 3, Fig. 3).

Our study findings shed light on physicians’ clinical practices and decision-making processes regarding GI motility disorders in Egypt; nearly half of the participants saw few patients with any GI motility disorders, less than 5 per week, or may not see one weekly. About one-third of them perform endoscopy, imaging at first, and then manometry when suspecting a GI motility disorder. About 24% may even try to treat and re-evaluate before imaging. More than 50% of them very frequently or consistently referred their suspected patient for endoscopy, and 47.9% for manometry. Unfortunately, only 26.9% of studied participants very frequently or consistently referred their suspected GI motility disorder patient to undergo a barium study, and only 13.8% were referred to psychiatry ((Table 4). The most frequent disorders that the participating physicians managed in their clinics were GERD and constipation, which is consistent with the global prevalence of constipation at fourteen percent [28], and the estimated global prevalence of GERD at 15%–25% [29].

When asked about having a motility unit/machine in their hospitals, more than half of the participants stated that they didn’t, in addition to 9.5% who didn’t know. They said this is due to financial problems or non-availability of experts. More than two-thirds of the studied participants paid attention. They were interested in participating in research concerning motility fields, stating that it is essential to diagnose GI motility disorders early. It is worth mentioning that most of the participants had a positive attitude towards GI motility practice. However, only 22.1% felt comfortable when dealing with a patient with a motility disorder. Most of the participating physicians were willing to attend training about motility disorders (96% for how to manage motility disorders medically, 94.6% for how to read a manometry report/topography, and 89.5% for investigations) rather than manometry [e.g., radiological] training (Table 5). This indicates potential gaps in resources and expertise within the healthcare infrastructure, suggesting a need for investment and capacity building to address GI motility disorders in Egypt better.

Standardized guidelines and improved coordination among healthcare providers are highlighted for patient care optimization. Training must be enhanced for suspecting, diagnosing, and managing motility disorders. Resource limitations, expertise boosts, and research initiatives are crucial in managing GI motility disorders in Egypt. The study reveals a lack of knowledge among Egyptian physicians about GI motility disorders, including symptom awareness and understanding of diagnostic modalities. Despite the lack of knowledge, most participants believe early diagnosis of GI motility disorders is important. Addressing knowledge deficits and improving physicians’ practice is essential for better patient outcomes in managing GI motility disorders. Training programs are needed to enhance physicians' knowledge and practice, covering the importance of medical and endoscopic management, early diagnosis, and various investigative modalities. Further research is required to evaluate training programs' effectiveness and identify barriers to GI motility practice in different regions. Studies should be conducted in other countries to understand physicians’ knowledge and practices regarding GI motility disorders.

### Limitations

Self-reported questionnaires may cause recall bias. The study had a diverse pool of physicians with different clinical backgrounds, expertise, and specialties, which can affect the level of data interpretation. Despite these challenges, the study aimed to study various responses, reflecting Egyptian physicians’ knowledge, attitudes, and practices about GI motility disorders.

## Conclusions

This study provides important insights into the current state of knowledge, practice, and attitudes of physicians regarding GI motility disorders in Egypt. The study highlights the urge for more training and educational programs to improve awareness among physicians regarding GI motility disorders. To achieve proper diagnosis and management of patients with GI motility disorders.

### Supplementary Information

Supplementary Material 1.

## Data Availability

The study data can be made available upon request from the primary or corresponding author.

## Abbreviations

GI: Gastrointestinal
SD: Standard deviation
CT: Computed tomography
GERD: Gastroesophageal reflux disease
POEM: Peroral Endoscopic Myotomy
APC: Argon plasma coagulation
