# Supplementary material for: Insights into the current state of knowledge, practice, and attitudes of physicians regarding gastrointestinal motility disorders in Egypt
Source: BMC Gastroenterol. 2024 Jul 4;24:215. doi: 10.1186/s12876-024-03296-7 (PMC11225201; doi:10.1186/s12876-024-03296-7)
Supplement: Supplementary file 1 — Supplementary Material 1. [file 12876_2024_3296_MOESM1_ESM.pdf]

1. Description

We are a group of Egyptian researchers from Ain Shams University and National Liver Institute, conducting a study about the "GI motility disorders ; What medical practitioners are aware of ?

• All physicians are invited to fill this questionnaire.
• It will take between 7-10 minutes to fill this questionnaire.
• Kindly answer all the questions by selecting what you believe is the best choice.
• Participation in this research is voluntary, and all information is anonymous and will be used only for research purposes.
• The participant can quit the research at any time.
• By agreeing to complete this form, you permit us to use the data in our research project.
• For any inquiries/questions kindly contact Dr. Enaam Ali
Email: drenaamali@yahoo.com

* 1. Do you agree to participate in this questionnaire?

◯ Yes

◯ No

2. Demographic Data

* 2. Gender

- Male
- Female

* 3. Age

* 4. Specialty

- Gastroenterology
- Internal Medicine
- GIT Surgery
- Pediatric Surgery
- General Surgery
- Geriatrics
- Other (please specify)

* 5. Last academic degree

- MBBch
- Diploma
- M.Sc.
- MD/PhD
- Fellowship
- Other (please specify)

* 6. Place of work

- University hospital
- Educational hospital
- Public hospital
- Research institute
- Private hospital/Clinic
- Military hospital
- Other (please specify)

* 7. Type of Health center working in

- ◯ Primary
- ◯ Secondary
- ◯ Tertiary hospital

* 8. Years of experience in your specialty

* 9. Level of experience in Endoscopy

- ☐ Diagnostic Upper GI Endoscopy
- ☐ Diagnostic Colonoscopy
- ☐ Therapeutic endoscopy
- ☐ Endoscopic retrograde cholangiopancreatography [ERCP]
- ☐ Endoscopic ultrasound [EUS]
- ☐ Not applicable

* 10. Years of experience in Endoscopy

* 11. In what country do you work?

* 12. If Egyptian, What is your area of work

- ◯ Upper Egypt
- ◯ Lower Egypt (Delta region and North Egypt)
- ◯ Greater Cairo
- ◯ Canal region and Sinai
- ◯ Other (please specify)

13. Contacts (Optional):

Name

Email Address

Phone Number

3. Knowledge

* 14. Do you know what GI motility studies are?

- ◯ Yes
- ◯ No

* 15. Which symptoms make you to suspect an upper GIT motility disorder?

	Symptom of upper motility disorder	Not a Symptom of upper motility disorder	Don’t Know
Dysphagia	◯	◯	◯
odynophagia	◯	◯	◯
Reflux symptoms	◯	◯	◯
Non cardiac chest pain	◯	◯	◯
Vomiting	◯	◯	◯
Eructation	◯	◯	◯
Hematemesis	◯	◯	◯
Epigastric pain	◯	◯	

◯ Other (please specify)

* 16. Which symptoms make you suspect a lower GI motility disorder?

	Symptom of lower GIT motility disorder	Not a symptom of lower GIT motility disorder	Don’t Know
Constipation	◯	◯	◯
Bloating	◯	◯	◯
Diarrhea	◯	◯	◯
Bloody diarrhea	◯	◯	◯
Incontinence	◯	◯	◯
Need to strain	◯	◯	◯
Sense of obstruction	◯	◯	◯
Long period between motions	◯	◯	◯

Other (please specify)

* 17. Grade the following investigation modalities regarding their priority in diagnosing different GI motility disorders.

	Strongly Agree	Agree	Neutral	Disagree	Strongly disagree
Endoscopy (Upper/Lower)	◯	◯	◯	◯	◯
Manometry	◯	◯	◯	◯	◯
pH study	◯	◯	◯	◯	◯
Barium study	◯	◯	◯	◯	◯
Scintigraphy	◯	◯	◯	◯	◯
Computed tomography of chest, abdomen & pelvis	◯	◯	◯	◯	◯
Endoscopic Ultrasound	◯	◯	◯	◯	◯

* 18. Do you have a motility unit/machine in your hospital?

- ◯ Yes
- ◯ No

19. If the previous answer is no, why?

- ◯ Of no use
- ◯ Financial
- ◯ No available experts
- ◯ Not needed as there is a nearby unit
- ◯ Other (please specify)

4. Practice

* 20. How frequent do you see patients with any GI motility disorders?

- <1 per week
- 1-5 per week
- 6-10 per week
- 11-15 per week
- >15 per week
- Not Applicable
- Other (please specify)

* 21. From your practice of view, what do you do when you suspect a GI motility disorder

- Refer to a gastroenterologist
- Perform endoscopy and imaging at first and then manometry
- Treat and re-evaluate
- Ask for manometry first
- Other (please specify)

* 22. How frequently do you refer patients for these interventions if you suspect a GI motility disorder (achalasia and gastroparesis)?

	Always	Very Frequently	Occasionally	Rarely	Never
Endoscopy					
Barium study					
Manometry					
Surgery					
Psychiatry					
Biofeedback (In cases of defecatory disorders/constipation)					

* 23. How frequent have you managed medically any of the following conditions?

	<1 case/month	1-5 cases/month	6-15 cases/month	16-30 cases/month	>30 cases/month
Achalasia	○	○	○	○	○
Nutcracker Esophagus	○	○	○	○	○
GERD	○	○	○	○	○
Rumination Syndrome	○	○	○	○	○
Gastroparesis	○	○	○	○	○
Constipation	○	○	○	○	○
Fecal incontinence	○	○	○	○	○

* 24. How frequently do you refer patients for these Interventions if you suspect motility disorder (achalasia , gastroparesis)?

	Always	Very Frequently	Occasionally	Rarely	Never
Peroral Endoscopic Myotomy (POEM)	○	○	○	○	○
Botulinum toxin injection	○	○	○	○	○
Stenting	○	○	○	○	○
Dilatation	○	○	○	○	○
Argon plasma coagulation (APC)	○	○	○	○	○

5. Attitude

* 25. Are you interested to participate in research studies in the field of motility studies?

- Yes
- No
- Not Sure

* 26. Do you think it is important to diagnose GI motility disorders?

- Yes
- No
- Not sure

* 27. Do you think that the patient will benefit from early diagnosis of a GI motility disorders?

- Yes
- No
- Not Sure

28. If the previous answer is no, Why?

* 29. What do you think the barriers for GI motility practice?

	Strongly Agree	Agree	Neutral	Disagree	Strongly disagree
Difficult branch or not well understood					
Lack of experts					
No available investigation tool					
Expensive investigations					
To the doctors it is not financially rewarding like endoscopic procedures					

Other (please specify)

* 30. How comfortable you are when dealing/reading manometry report?

- Very comfortable
- Comfortable
- Neutral
- Not comfortable
- Not comfortable at all
- Not Applicable (I don’t receive any reports)

* 31. How comfortable you are when dealing with a patient with motility disorder?

- Very comfortable
- Comfortable
- Neutral
- Not comfortable
- Not comfortable at all
- Not Applicable

* 32. If there will be training about motility disorders, what you would like it to be about:

	Agree	Disagree
How to suspect and diagnose motility disorders.	○	○
How to manage motility disorders ○ medically	○	
Endoscopic management of motility disorders	○	○
How to read a manometry report/ ○ topography	○	
Investigations rather than manometry (e.g., radiological)	○	○

Other (please specify)
